# Altered AKT1 and MAPK1 Gene Expression on Peripheral Blood Mononuclear Cells and Correlation with T-Helper-Transcription Factors in Systemic Lupus Erythematosus Patients

**DOI:** 10.1155/2012/495934

**Published:** 2012-10-18

**Authors:** Sonia Garcia-Rodriguez, Jose-Luis Callejas-Rubio, Norberto Ortego-Centeno, Esther Zumaquero, Raquel Ríos-Fernandez, Salvador Arias-Santiago, Pilar Navarro, Jaime Sancho, Mercedes Zubiaur

**Affiliations:** ^1^Department of Cellular Biology and Immunology, Instituto de Parasitología y Biomedicina López-Neyra, (IPBLN-CSIC), Parque Tecnológico Ciencias de la Salud, Avenida Conocimiento s/n, Armilla, 18100 Granada, Spain; ^2^Systemic Autoimmune Diseases Unit, Department of Internal Medicine, San Cecilio University Hospital (SCUH), Avenida Dr. Oloriz, no. 16, Granada 18012, Spain; ^3^Department of Microbiology, University of Alabama at Birmingham, 1720 2nd Ave South, Birmingham, AL 35294, USA; ^4^Department of Dermatology, SCUH, Avenida Dr. Oloriz, No. 16, Granada 18012, Spain

## Abstract

Kinases have been implicated in the immunopathological mechanisms of Systemic Lupus Erythematosus (SLE). v-akt murine-thymoma viral-oncogene-homolog 1 (AKT1) and mitogen-activated-protein-kinase 1 (MAPK1) gene expressions in peripheral mononuclear cells from thirteen SLE patients with inactive or mild disease were evaluated using quantitative real-time reverse-transcription polymerase-chain-reaction and analyzed whether there was any correlation with T-helper (Th) transcription factors (TF) gene expression, cytokines, and S100A8/S100A9-(Calprotectin). Age- and gender-matched thirteen healthy controls were examined. AKT1 and MAPK1 expressions were upregulated in SLE patients and correlated with Th17-(Retinoic acid-related orphan receptor (ROR)-C), T-regulatory-(Treg)-(Transforming Growth Factor Beta (TGFB)-2), and Th2-(interleukin (IL)-5)-related genes. MAPK1 expression correlated with Th1-(IL-12A, T-box TF-(T-bet)), Th2-(GATA binding protein-(GATA)-3), and IL-10 expressions. IL-10 expression was increased and correlated with plasma Tumor Necrosis Factor (TNF)-**α** and Th0-(IL-2), Th1-(IL-12A, T-bet), GATA3, Treg-(Forkhead/winged-helix transcription factor- (FOXP)-3), and IL-6 expressions. FOXP3 expression, FOXP3/RORC, and FOXP3/GATA3 expression ratios were increased. Plasma IL-1**β**, IL-12(p70), Interferon-(IFN)-**γ**, and IL-6 cytokines were augmented. Plasma IL-1**β**, IL-6, IL-2, IFN-**γ**, TNF-**α**, IL-10, and IL-13 correlated with C-reactive protein, respectively. Increased Calprotectin correlated with neutrophils. Conclusion, SLE patients presented a systemic immunoinflammatory activity, augmented AKT1 and MAPK1 expressions, proinflammatory cytokines, and Calprotectin, together with increased expression of Treg-related genes, suggesting a regulatory feedback opposing the inflammatory activity.

## 1. Introduction

Systemic Lupus Erythematosus (SLE) is a chronic inflammatory autoimmune disease most common in women of reproductive age [1], characterized by a relapsing/remitting course and the involvement of multiple organs, including skin, kidneys, and central nervous system. Pathophysiologically is characterized by the dysfunction of T, B, and dendritic cells (DC), skewed cytokine production, breakdown of immunological tolerance, and by the production of antinuclear autoantibodies [2–5]. 

In SLE bone marrow mononuclear cells, Nakou et al. [6] identified central gene regulators implicated in disease pathogenesis which include activation of multiple kinase pathways (MAPK/extracellular regulated MAP kinase (ERK), Signal Transducer and Activator of Transcription (STAT), AKT, and PI3-kinase (PI3 K)). AKT1 serine/threonine kinase is a key downstream target of PI3 K signaling pathway, and plays a role in the differentiation of peripheral B cells and in T cell homeostasis [7–10]. Upregulated activity of AKT kinases has been documented in B cells from SLE patients [11]. AKT/Mammalian target of rapamycin (mTOR) axis has been successfully targeted with rapamycin for treatment of SLE patients [2]. MAPK/ERK kinases play a significant role in immune-mediated inflammatory responses and are involved in the maintenance of T-cell tolerance that fails in SLE patients [4, 12]. Molad et al. [13] reported increased ERK and JNK activities that correlated with disease activity in SLE patients. Conversely, other studies documented a reduced MAPK activity in human SLE T cells [4]. In mice, a defect in lupus T cell ERK pathway signaling that can cause epigenetic abnormalities in T cells by inhibiting DNA methylation has been reported [12, 14]. In addition, in SLE T cells, alterations in kinases pathways such as the spleen tyrosine kinase (Syk) signaling patterns have been documented by Krishnan et al. [15].

The imbalance in cytokine activities is associated with human autoimmune and autoinflammatory diseases, and it has been reported to play a central role in regulating SLE development [16–20]. There is a key collaboration and regulation between key cytokines in activation/induction of transcription factors during the process of T-helper-(Th)-cell differentiation towards Th1, Th2, Th17, and induced regulatory T (iTreg) cells lineages [21, 22]. Changes in Th-specific transcription factors gene expression ratios have been used as a marker of Th-cytokine balance [20, 23–26]. 

Moreover, increased inflammation mediators S100A8/S100A9-(Calprotectin) serum and/or plasma levels have been reported in SLE patients [27–29]. Damage-associated molecular pattern molecules (DAMP) S100A8 and S100A9 members of the calcium-binding S100-family make a heterotetrameric form, S100A8/S100A9-(Calprotectin). 

The aim of this study is to assess the gene expression levels of intracellular kinases AKT1 and MAPK1 in peripheral blood mononuclear cells (PBMC) from SLE patients with inactive or mild disease and to analyze whether there was any correlation with Th-transcription factors gene expression, with gene expression and plasma levels of a comprehensive panel of cytokines (Th0-, Th1/Th17-, and Th2/Treg-type), with plasma inflammation mediators S100A8/A9-Calprotectin and clinical parameters.

## 2. Materials and Methods

### 2.1. Patients and Controls

The study protocol was approved by the SCUH Committee and CSIC Review Board and Ethics Committees. Written informed consent was obtained from all participating patients and controls, according to the Helsinki Declaration. Clinical data and treatments of patients are summarized (Table 1). Thirteen SLE outpatients (eleven women and two men) with inactive or mild disease (SLE disease activity index (SLEDAI) score ≤ 4) (0 (0–3) (median (25% and 75% percentile ranges), see Table 1) that fulfilled at least four of the revised SLE criteria of the American College of Rheumatology (ACR) for the diagnosis of SLE [30, 31] were evaluated. Age- and gender-matched healthy controls (C) included thirteen volunteers subjects, without known autoimmune disease (eight women and five men; 34 (30–51.5) years old; differences versus SLE patients were not statistically significant, Mann-Whitney, *P* = 0.1435). Likewise, no significant differences in gender distribution were observed between patients and controls (Fisher test: *P* = 0.3783, for SLE patients versus C). Patients and controls were Caucasians. 

Medications taken by the patients at the time the blood was drawn were recorded (Table 1). The majority of SLE patients (*n* = 12) were treated with hydroxychloroquine (200 mg/day); nine SLE patients were also treated with prednisone (5 mg/day), and six of them were additionally treated with one of methotrexate, azathioprine, or mycophenolate mofetil (Table 1). 

### 2.2. Blood, Plasma, and PBMC Samples

Blood was collected (BD Vacutainer system, K2-EDTA tubes, BD Diagnostics, Franklin Lakes, NJ, USA) and plasma and PBMC were separated using density gradient centrifugation [24].

### 2.3. RNA Isolation and Quantitative Real-Time Reverse Transcription-Polymerase Chain Reaction (qRT-PCR)

Gene expression levels were measured quantitatively by qRT-PCR as previously described [24]. Total RNA was isolated from PBMC from thirteen SLE patients, and four controls, using RNeasy Plus mini kit (Qiagen, Hilden, Germany). RNA quality was assessed by an Experion Automated Electrophoresis Station (Bio-Rad, Hercules, CA, USA). RT reaction, primer sequences, and gene-specific primers used in this studio are indicated (Table 2 and Supplementary Materials). Glyceraldehyde-3-phosphate dehydrogenase (GAPDH) was used as an endogenous control. To quantify cytokine mRNA expression, a RT^2^ Profiler PCR Array System (SABiosciences, Qiagen) was used. The PCR Array layout contained gene-specific primers for ten cytokines: Interleukin (IL)-1B, IL-2, IL-5, IL-6, IL-10, IL-12A, Tumor Necrosis Factor (TNF)-*α*, Interferon (IFN)-*γ*, Transforming Growth Factor Beta (TGFB)-2, and Tumor Necrosis Factor-Related Apoptosis-Inducing Ligand or TRAIL (TNFSF)-10; two internal loading gene-specific primers controls were included for standardization between samples (GAPDH and *β*-actin, Table 2). Proportion of transcript present in the samples was calculated using the relative quantification 2^−ΔΔCt^ scheme [32]. Control samples were used as comparative calibrator. Results represented the relative amount of amplicon in patient's sample (fold change) to the mean level of the transcripts in the control samples.

### 2.4. Plasma Cytokines

Bio-Plex Precision Pro Human Cytokine 10-Plex kit assays were used to simultaneously test 10 cytokines in plasma: IL-1*β*, IL-2, IL-4, IL-5, IL-6, IL-10, IL-12(p70), IL-13, IFN-*γ*, and TNF-*α*, in according with manufacturer's protocol (Bio-Rad). 

### 2.5. Human S100A8/S100A9 Heterodimer Complexes or Calprotectin Enzyme Linked Immunosorbent Assay (ELISA)

Plasma concentration, a noncovalently associated Calprotection, in the presence of calcium, was measured in duplicate using a sandwich ELISA kit (no. HK325; Hycult Biotechnology, Uden, The Netherlands).

### 2.6. Statistics

Data are given as median and 25% and 75% percentile ranges, or otherwise indicated. Relative mRNA expression levels of the genes in patients' PBMC versus controls were evaluated using the nonparametric Wilcoxon Signed Rank test and comparing the medians against a hypothetical median (value equal 1). Determinations of significant differences between groups were compared using the non-parametric Mann-Whitney *U* test. Fisher exact test was used for analysis of qualitative variables. Correlations were determined by Spearman's rank correlation test. Differences were considered statistically significant for *P* values < 0.05 (GarphPad Prism v.5.01, GraphPad, CA, USA).

## 3. Results

### 3.1. Intracellular kinases and Immunoreceptors Gene Expressions Levels

**Figure 1:**
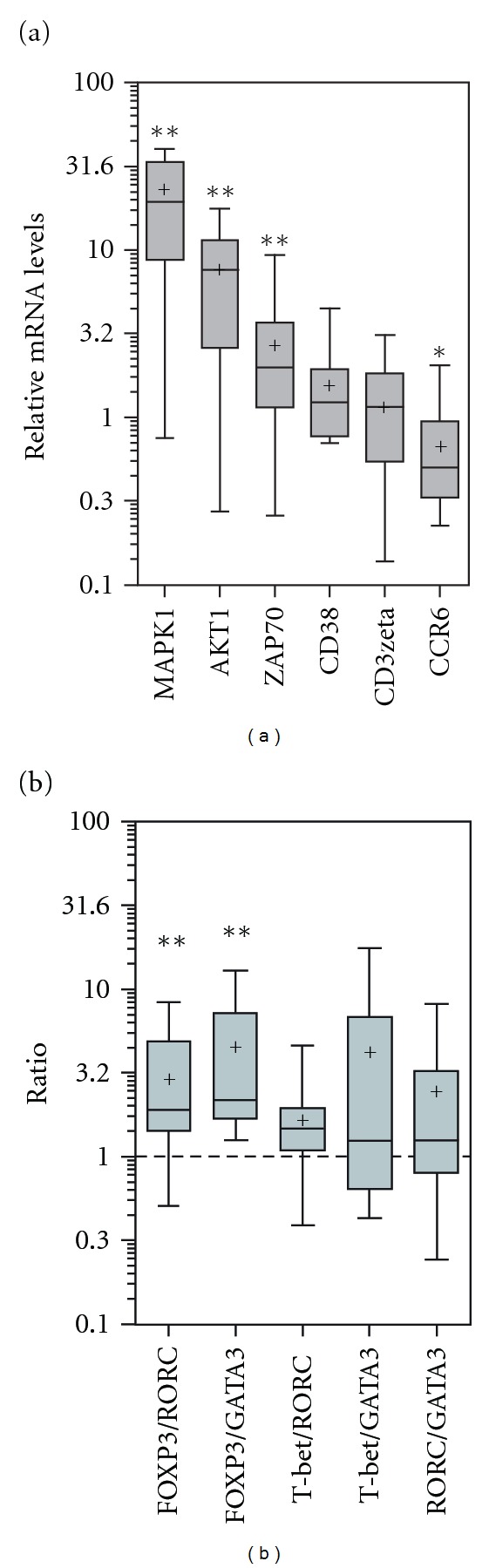
(a) Intracellular kinases and immunoreceptors gene expressions levels. RNAs extracted from PBMC of SLE patients (*n* = 13) and controls (*n* = 4) were reverse transcribed, and the gene expression levels were determined by qRT-PCR using gene-specific primers for indicated genes and GAPDH to normalize the mRNA expression (Materials and Methods, Supplementary Materials, and Table 2). Proportion of transcript present in the samples was calculated using the relative quantification 2^-ΔΔCt^ scheme. Control samples were used as comparative calibrator. Final results represent the relative amount of amplicon in patient's sample (fold change) to the mean level of the transcripts in the control samples. In the figure, results are represented in box plots given the median (horizontal bars within boxes), the mean (cross points); box limits correspond to quartiles, and vertical lines indicate the 5–95 percentile range. Results are represented in a log 10 scale, and the indicated numbers are the antilog values. Significance levels set at *P* values <0.05. AKT1, MAPK1, and ZAP70 gene expressions were increased in SLE patients compared with controls (Wilcoxon Signed Rank test: *P* = 0.0064, *P* = 0.0012, *P* = 0.0024). Gene expression of CCR6 was decreased in SLE patients compared with controls (Wilcoxon Signed Rank test: *P* = 0.0398). (b) T-helper transcription factors gene expression ratios in SLE patients. Results are expressed as indicated in (a). FOXP3/RORC ratio in SLE patients versus controls (Wilcoxon Signed Rank test, *P* = 0.0042). FOXP3/GATA3 ratio in SLE versus controls (Wilcoxon test, *P* = 0.0017).

AKT1 (7.82 (2.61–11.45)), MAPK1 (19.47 (8.71–33.45)), and ZAP70 (1.98 (1.14–3.66)) relative gene expressions levels were significantly augmented in PBMC from SLE patients (*n* = 13) as compared with controls (Wilcoxon Signed Rank test: *P* = 0.0064, *P* = 0.0012, and *P* = 0.0024, resp.) (Figure 1(a)). CD38 (1.22 (0.77–1.95)) and CD3zeta (1.15 (0.54–1.83)) immunoreceptors relative gene expression levels were also evaluated; not significantly differences were observed versus controls (Wilcoxon Signed Rank test: *P* = 0.0805, *P* = 0.7869, resp.) (Figure 1(a)). In addition, the Th17-associated CC chemokine receptor (CCR)-6 relative gene expression was significantly decreased in SLE's PBMC versus controls (0.50 (0.33–0.95), Wilcoxon Signed Rank test, *P* = 0.0398) (Figure 1(a)). 

### 3.2. Increased Treg/Th17 and Treg/Th2 Transcription Factors Gene Expression Ratios

In PBMC from patients and controls, relative gene expression of T-box transcription factor (T-bet or TBX21) and signal transducer and activator of transcription (STAT)-4, both for Th1 cells; GATA3, a member of the GATA family of zinc finger proteins for Th2; retinoic acid-related orphan receptor (ROR)-C, for Th17 cells and forkhead/winged helix transcription factor (FOXP)-3 for Treg cells [33, 34] were assessed and Th-transcription factors gene expression ratios calculated. FOXP3 relative gene expression was significantly increased in SLE patients versus controls (18.3 (1.39–44.14), *n* = 13, Wilcoxon test, *P* = 0.0012). However, there were no statistically significant differences between medians of the T-bet (5.94 (0.44–63.98)), STAT4 (1.09 (0.60–2.07)), RORC (4.49 (0.60–30.56)) and GATA3 (2.80 (0.79–8.19)) relative gene expressions levels, respectively, versus controls (Wilcoxon Signed Rank test, *P* = 0.0681; *P* = 0.3054, *P* = 0.0681, *P* = 0.0574, for T-bet, STAT4, RORC, and GATA3, resp.).

In SLE patients, gene expression ratios of both FOXP3/RORC (1.90 (1.41–4.85)) and FOXP3/GATA3 (2.17 (1.67–7.22)) were significantly increased versus controls (Wilcoxon Signed Rank test, *P* = 0.0042 and *P* = 0.0017, resp.) (Figure 1(b)). While, ratios of T-bet/RORC (1.48 (1.09–1.95)), T-bet/GATA3 (1.24 (0.64–6.77)), and RORC/GATA3 (1.25 (0.79–3.23)) were evaluated and no significantly differences were observed between SLE versus controls (Wilcoxon Signed Rank test, *P* = 0.0503, *P* = 0.1099, and *P* = 0.0942, resp., Figure 1(b)). 

### 3.3. Cytokines Gene Expression, Plasma Levels, and Correlations

In SLE patients, cytokines mRNA expressions were evaluated using a RT^2^ Profiler PCR Array System. Results indicated that IL-10 relative gene expression was significantly increased versus controls (Table 3). In addition, ten cytokines were simultaneously assessed in each plasma sample using a Bio-Plex (Table 4). Results indicated that plasma levels of proinflammatory cytokines IL-1*β*, IL-6 (both Th17), IL-12(p70), IFN-*γ* (both Th1) together with IL-5 (Th2) were significantly augmented in SLE patients versus controls (Table 4). 

In SLE (*n* = 13) patients, IL-1*β* and IL-6 plasma levels were positively correlated to IL-1B and IL-6 gene expression levels, respectively (Table 5). IL-6 gene expression also positively correlated with Th1 (IL-12(p70), IFN-*γ*, and TNF-*α*), Th0 (IL-2), Th17 (IL-1*β*), and IL-10 plasma cytokines, respectively. There was a positive correlation between IL-10 gene expression and plasma TNF-*α* (Table 5). While there was a negative correlation between IL-10 and TNF-*α* gene expressions (*r* = −0.6905, *P* = 0.009). There were significant positive correlations in the IL-2, IL-6, IL-10, IL-12A, and TGFB2 gene expressions, which were correlated to one another; except for IL-6 and TGFB2 (Table 5). In addition, IFN-*γ* and IL-12A gene expressions were positively correlated (Table 5). 

### 3.4. AKT1, MAPK1, and Th-Transcription Factors Gene Expressions Correlations

In SLE patients, there were significant correlations between MAPK1, AKT1, T-bet, RORC, and GATA3 relative gene expressions, respectively (Table 6). Additionally, AKT1 gene expression was correlated to both RORC (Table 6) and CD38 expressions (Table 8), respectively. Moreover, there were significant correlations in the T-bet, STAT4, GATA3, RORC, and FOXP3 gene expressions, which were correlated to one another (Table 6). Both AKT1 and MAPK1 gene expressions were correlated with gene expression levels of Treg-(TGFB2) and Th2-(IL-5) cytokine-related genes, respectively (Table 7). There was also a correlation between AKT1 and TNFSF10 (TRAIL) gene expressions (Table 7). Additionally, MAPK1 gene expression was correlated to IL-10 and IL-12A gene expressions (Table 7). Furthermore, significant correlations between IL10 relative gene expression and FOXP3, RORC, GATA3, T-bet, (Table 7), and CD38 gene expressions (Table 8), respectively, were observed. Correlations between FOXP3 gene expression and Th1 (IL-12A, IFN-*γ*), Th0 (IL-2), Treg-(TGFB2), Th2-(IL-5), and IL-6 gene expressions, respectively, were observed (Table 7). 

### 3.5. Plasma S100A8/A9 Calprotectin, Clinical Parameters, and Correlations

Plasma levels of inflammation mediator Calprotectin were augmented in SLE patients (175.6 (138.9–229.2) ng/mL, *n* = 13), as compared with controls (143.3 (133.4–151.9) ng/mL, *n* = 11) although differences were not statistical significant (Mann-Whitney, *P* = 0.0637). Our data are consistent with previous reports [27–29, 35]. In SLE patients, Calprotectin was correlated with neutrophils numbers (Table 8). In addition, significant correlations were obtained between disease-marker C-reactive protein (CRP) and plasma cytokines type Th1-(IFN-*γ*, TNF-*α*), Th0-(IL-2), Th2-(IL-13), Th17-(IL-1*β*, IL-6), and IL-10, respectively (Table 8). Patients' age was correlated with gene expression levels of IL-2, IL-10, IL-12A, TGFB2, and GATA3 relative gene expressions, respectively (Table 8). Moreover, in SLE patients, C4-complement levels were correlated to CCR6 (lymphocyte trafficking) and STAT4 gene expressions levels, respectively, which were correlated to one another (Table 8). In mice, CCR6 was recently identified by Wei et al. [36] among the STAT4-occupied genes. Lastly, lymphocytes numbers negatively correlated with TNFSF10 (TRAIL) gene expression (Table 8). 

## 4. Discussion

In this study, we observed that AKT1 and MAPK1 gene expressions were upregulated in SLE patients with mild or inactive disease and correlate with gene expressions levels of Th17-(RORC), Treg-(TGFB2), and Th2-(IL-5)-related genes, respectively. Additionally, AKT1 gene expression also correlates with MAPK1 expression in SLE patients. PI3 K/AKT and MAPK pathways are regulated by extensive crosstalk at different levels [9, 37]. AKT effect on the generation and function of Treg and Th17 cells is not well understood. Wan et al. [38] reported that RORC was necessary, but not sufficient, for IL-17 production by human memory T cells, and they proposed that RORC synergized with common *γ* chain-cytokines mediated PI3 K and AKT signaling to transactivate IL-17 expression. Recently, Kurebayashi et al. [39] demonstrated in mice that the suppression of PI3 K-AKT-mammalian target of Rapamycin complex (mTORC)-1 axis impaired Th17 differentiation *in vitro* and *in vivo* in S6 kinases 1 and 2-dependent fashion. In mice, Sauer et al. [40] documented that T cell receptor (TCR) signaling via PI3Kp110*α*, p110*δ*, AKT, and mTOR controls FOXP3 expression in activated CD4^+^ thymocytes and peripheral T cells. PI3 K-AKT axis has also been reported augments clonal expansion of Th1 and Th2 cells [41, 42]. In mature T cells, constitutively active AKT could induce growth and survival of CD4^+^ [8]; and a direct role of AKT in CD8^+^ T cells has been reported, regulating whether their differentiation leads them to either memory or effector fate [10]. In SLE patients, Suarez-Fueyo et al. [43] reported that PI3 K pathway activation paralleled activated/memory T cell accumulation. Our results show in SLE patients that AKT1 expression positively correlates with TNFSF10 (TRAIL) expression, and TRAIL expression negatively correlates with lymphocytes numbers. MAPK/ERK and PI3 K/AKT signaling pathways are activated by TRAIL [44]. Up-regulation of TRAIL mRNA expression in PBMC from active SLE patients was reported [45]. TRAIL-induced apoptosis by binding to death receptors but also can enhance T cell proliferation after TCR engagement via transducing a costimulation signal [46]. 

Our results show that MAPK1 gene expression also correlates with gene expression levels of Th1-(IL-12A, T-bet), Th2-(GATA3), and IL-10 genes. MAPK/ERK signaling pathways integrate cytoplasmic signals to produce changes in transcription associated with differentiation, proliferation, and survival in multiple stages of T cell development [47–49]. The results show a clear increase of IL-10 relative gene expression in SLE PBMC, although IL-10 plasma levels are not significantly augmented. Meta-analysis of microarray data indicated that one of the biological pathways consistently enhanced in SLE patients was the IL-10 signaling [50]. IL-10 has been identified as one of the risk loci for SLE in a recent genome-wide association study [51]. Despite that no correlation of IL-10 gene expression with SLEDAI was observed, IL-10 expression positively correlates with plasma TNF-*α* (Table 8), and with expression levels of Th0-(IL-2), Th1-(IL-12A, T-bet), Th2-(GATA3), Treg-(TGFB2, FOXP3), pro-inflammatory IL-6, and Th17-(RORC)-cytokine related genes, respectively. IL-10 is a key cytokine regulating immune responses, and the absence results in spontaneous inflammatory disorders as well as exacerbated inflammation in T cell responses during host defense or autoimmune inflammation (see reviews [52, 53]). IL-10 is expressed by cells of the adaptive immune system (Th1, Th2, Treg cells, and regulatory B cells (Bregs) and by cells of the innate immune system (DCs, macrophages, mast cells, NK, eosinophils, and neutrophils) [54, 55]. IL-10 provides a highly regulated feedback loop to avoid the extremes of excessive inflammation or chronic infections and also allow a protective response to pathogens [54]. In mice, Saraiva et al. [56] reported that development of IL-10-producing Th1 cells required high TCR ligation, sustained ERK1 and ERK2 MAPKs phosphorylation, and IL-12-induced STAT4 activation, indicating that ERK1 and ERK2 activation was a common pathway required for the production of IL-10 by Th1, Th2, and Th17 cell subsets. Regulation of IL-10 expression in mouse NK cells reported by Grant et al. [57] indicated that like IFN-*γ*, IL-10 expression was induced by IL-2 and IL-12 stimulation. Cytokines like TGF-*β* and IL-6 are also important regulators of IL10-producing cells [58]. It has been reported by Avagyan et al. [59] that TGF-*β*2 specifically enhanced signaling by the hematopoietic growth factor fms-related tyrosine kinase (FLT)-3 in hematopoietic stem cells. In activated human T cells, Astier et al. [60] documented that FLT3 growth factor was acting as a negative regulator of IL-10 levels. Furthermore, a relevant role of IL-10 and TNF-*α* genotypes in SLE has been reported [61], and IL-10 cytokine inhibits transcription elongation of the human TNF gene in primary macrophages [62]. Our results also show that IL-10 gene expression in SLE patients correlates with CD38 (activation, maturation, and trafficking marker) gene expression; CD38 expression correlates with AKT1 expression. In human T and B cells, upon receptor stimulation CD38 leads to PKB/AKT and ERK activation [63, 64]. Moreover, in a kinase-dead mutant of PI3Kp110*δ* mice, Patton et al. [65] reported that PI3Kp110*δ* kinase regulates expression of CD38 on Treg. Furthermore, Blair et al. [66] documented that CD19^+^CD24^hi^CD38^hi^-B cells exhibit regulatory capacity in healthy individuals while the same B cells from SLE patients produced less IL-10 and lacked the suppressive capacity.

Our data show a clear increase in FOXP3-(Treg) gene expression levels in SLE patients and positively correlate with IL-10 gene expression. Recent reports provided new data about the mechanisms linking IL-10 and Treg cells by demonstrating that IL-10 directly signals in Th17 and Treg cells to maintain control of Th17 cell-mediated inflammation [67, 68]. In addition, IL-10-producing B cells, also known as regulatory B cells (Bregs), play a key role in controlling autoimmunity. Carter et al. [55] reported that chimeric mice specifically lacking IL-10-producing B cells developed an exacerbated arthritis compared with chimeric wild-type B cell mice. Moreover, a significant decrease in the absolute numbers of FOXP3 regulatory T cells (Tregs), in their expression level of FOXP3 and a marked increase in inflammatory Th1 and Th17 cells were detected in chimeric mice lacking IL-10-producing B cells compared with WT B-cell mice [55].

How Treg cell function and FOXP3 expression are regulated is an important question under intensive investigation [69]. Barreto et al. [70] reported a promoter polymorphism in FOXP3 gene associated with SLE. In SLE patients, increased gene expression of FOXP3 compared to healthy controls has been reported [71]. In SLE patients, several reports indicated that increased proportions of CD4^+^CD25^−^Foxp3^+^ T cells were correlated with the clinical disease activity and the daily cortisone dose [72, 73]. In our study, we cannot exclude that treatments received by SLE patients could have an effect on the results. Although, a recent report from Sbiera et al. [74] documented that short-term glucocorticoid therapy *in vivo* either in immunologically uncompromised humans or in mice did not induce an increased in the frequency of circulating Treg cells. 

Balance between FOXP3 and RORC function has been postulated that determines CD4^+^T cell fate and the type of immune response that will be generated [75]. Th17 and Treg seem to be closely linked [33, 76]. Several studies reported in active SLE patients an imbalance between Treg and Th17 cells [77–79]. Moreover new information has been reported about the interplay between Tregs and Th2 cells [80]. Wang et al. [81] demonstrated an essential role for GATA3, in controlling Treg cell homeostasis by stabilizing expression of FOXP3. Our results show an increased balance of both FOXP3 (Treg)/RORC (Th17) and FOXP3 (Treg)/GATA3 (Th2) gene expression ratios in PBMC from SLE patients with inactive or middle disease, suggesting a trend towards Treg polarization. In addition, our results show no significant increases compared with controls in the T-bet (Th1)/RORC (Th17), T-bet (Th1)/GATA3 (Th2), and RORC (Th17)/GATA3 (Th2) gene expression ratios in PBMC from SLE patients. Our data also show a low relative gene expression levels of Th17-associated CCR6 (is a trait of tissue-homing effector T cells lymphocyte trafficking) in PBMC from the SLE patients with inactive or middle disease. CCR6 expression on CD4^+^T cells has been considered as a marker of disease activity in SLE patients [82]. In addition, Sallusto et al. [83] reported that following TCR stimulation, human memory/effector T cells down-regulate receptors for constitutive chemokines (including CCR6). They suggested that following TCR stimulation effector/memory T cells transiently acquire responsiveness to constitutive chemokines. As a result, T cells that are activated in tissues may either recirculate to draining lymph nodes or migrate to nearby sites of organized ectopic lymphoid tissues [83]. 

Several studies have shown a trend towards increased Th17 cells and Th17/Th1 ratio in SLE patients with active disease [82, 84]. Furthermore, reports on function and numbers and Treg cells in SLE have been contradictory (reviews [75, 85]), suggesting that not all FOXP3^+^T cells in SLE have protective suppressive activity. Plasticity of CD4^+^FOXP3^+^T cells has been documented [34, 86], and it has been reported that conversion of human FOXP3^+^ Tregs into IL-17-producing cells can be enhanced in the context of an inflammatory cytokine milieu (IL-1*β* and IL-6) [75, 76, 86, 87].

Tregs presented core suppressive mechanisms driven by FOXP3. Nevertheless they are also able to adapt in response to stimuli by homing to sites of inflammation and exerting suppressive functions. It has been reported that Treg are able to express other transcription factors normally associated with other Th cell subtypes in order to better control immunopathology (see review [34]). In our study, plasma levels of proinflammatory cytokines IL-1*β*, IL-12(p70), IFN-*γ*, and IL-6 are significantly augmented in SLE patients with inactive or middle disease compared with controls. In SLE it has been suggested that aberrant control of immune cell responses to inflammatory cytokines may disrupt the delicate balance between immunity and self-reactivity [2, 3, 88]. IL-6 has been studied in human SLE, and IL-6 was found to reflect disease activity [19, 89–93]. IL-6 contributes to host defence against acute environmental stress, and continuous deregulated IL-6 production plays a significant pathological role in systemic autoimmune diseases [94]. IL-6 has been known to have a key role in the maturation of B cells, as well to be one of the members of cytokines that drives the acute inflammatory response together with TNF-alpha and IL-1 [94]. Additionally, plasma IL-5 (Th2) was significantly augmented in SLE patients versus controls. IL-5 is produced by natural helper cells and is involved in antibody production [95, 96]. IL-5 is a critical growth factor for B1 cells present in the peritoneal cavity and plays an important role in innate-type immune responses by producing natural antibodies [97–99]. Wen et al. [100] reported that deregulated, continuous high expression of IL-5 in SLE-prone mice may directly or indirectly mediate a skewed signaling of proliferation/differentiation of self-antigen-activated B1 cells, leading to suppression of autoimmune disease, but instead of aberrant expansion of B1 cells, giving rise to B-cell chronic lymphocytic leukaemia (B-CLL). Moreover, despite no correlation with SLEDAI, our results indicate that Th17-(IL-1*β*, IL-6), Th0-(IL-2), Th1-(IFN-*γ*, TNF-*α*), Th2-(IL-13), and IL-10 plasma cytokines are positively correlated with the inflammation mediator CRP. During inflammation altered cytokine levels are produced by different cell populations in an interdependent mode [101]. These results are consistent with those of other studies [102, 103].

Moreover, our results show a moderate increase of ZAP70 tyrosine kinase gene expression in SLE's PBMC and ZAP70 correlate with gene expressions levels of TNF-*α* (Table 8). Syk and ZAP-70 kinases also associated differently with key molecules involved in cytoskeletal and calcium signaling in SLE T cells [15]. In human mature T cells, Meinl et al. [104] demonstrated a key role of ZAP70 in CD2-mediated phosphorylation of CD3zeta chain of TCR, in Ca^2+^ influx, proliferation, and production of IFN-*γ* and TNF-*α*. 

Furthermore our data show that increased inflammation mediator S100A8/S100A9-Calprotectin plasma levels in SLE patients positively correlate with neutrophils numbers. S100A8/S100A9-Calprotectin is expressed by neutrophils, monocytes, activated macrophages [105] and released in Neutrophil Extracellular Traps [106]. These results also accord with our earlier observations [29], which showed in SLE PBMCs that increased S100A9 levels, correlated positively with the abnormal presence of low-density granulocytes detected by flow cytometry in the mononuclear cells. 

Despite the fact that the study has been performed in a small number of patients, the results and the positive correlations found suggest biological functions and associations that need to be further confirmed in a larger cohort of patients. 

## 5. Conclusions 

SLE patients with inactive or mild disease presented a systemic immunoinflammatory activity with augmented AKT1 and MAPK1 gene expressions, plasma proinflammatory cytokines, and Calprotectin, together with increased gene expression of Treg-related genes, suggesting an ongoing regulatory feedback opposing the inflammatory activity.

## Supplementary Material

qRT-PCR procedures: Reverse transcription (RT) reaction was performed on 1 *µ*g total RNA, 20-*µ*l reaction, containing 200 U SuperScript III reverse transcriptase (Invitrogen), 5 mM random hexamer primers, 0.5mM each deoxyribonucleotide triphosphate (dNTP), 20 U RNase inhibitor, and 1X-RT buffer. Primer sequences and gene-specific primers used are available in Table 2. Gene expressions assays were performed on iQ-Cycler (Bio-Rad), with 3 replicates with iQ-SYBR Green Supermix, 21-microliter reaction (200nM each primer and 10 ng of cDNA). PCR program: 10 min incubation at 95°C, 40 cycles of 15 s at 95°C, and 1 min at 60°C. Specificity of PCR amplification procedure was checked with a heat dissociation protocol (from 65°C to 100°C), after the final cycle of the PCR.

## Figures and Tables

**Table 1:** Clinical parameters and treatments of SLE patients.

SLE patients	SLEDAI^a^	Sex^b^	Age^c^	L^d^	C3^e^	C4^e^	Anti-dsDNA^g^	RD^h^	CRP^i^	N^j^	Dd^k^	Treatment^l^
1	4	W	41	1620	93	17	−	−	0.11	4670	3.67	HCQ
2	0	W	27	1470	108	19	−	−	0.07	9870	0.75	HCQ/PN
3	0	W	65	2530	112	21	−	−	0.17	4760	4.5	HCQ/PN
4	0	W	64	2510	95	25	−	−	0.1	7130	10.5	HCQ
5	4	W	43	900	73	6	+	−	1.13	6870	8.67	HCQ/PN
6	2	M	59	2240	88	8	−	−	4.62	8240	1.08	MTX/PN/HCQ
7	0	W	56	1130	131	20	−	−	0.05	4070	2.58	NT
8	4	W	42	570	66	9	+	+	0.01	3120	8.83	AZA/PN/HCQ
9	0	W	40	750	62	12	+	−	1.16	7500	8.83	MTX/HCQ/PN
10	0	W	55	1740	110	15	−	−	0.35	8250	2.33	MMF/PN/HCQ
11	2	M	18	3420	129	11	+	+	ND^f^	12260	5.33	MMF/PN/HCQ
12	2	W	35	2250	91	23	−	+	0.08	6190	19.25	AZA/HCQ/PN
13	0	W	41	1150	ND^f^	ND^f^	−	−	0.7	3170	6.75	HCQ

Median^m^	0		42	1620	94^n^	16^n^			0.14^n^	6870	5.33	
	(0–3)		(37.5–57.5)	(1015–2380)	(76.8 –111.5)	(9.5– 20.8)			(0.072–1.023)	(4370–8245)	(2.5–8.8)	

^a^Systemic Lupus Erythematosus Disease Activity index (SLEDAI).

^b^W: woman; M: man.

^c^Age (years).

^d^L: Lymphocytes/mL.

^e^Complement C3 and C4 (low <90 and/or <10, mg/dL).

^f^ND: no determined.

^g^SLE patients presented anti-double stranded DNA auto antibodies (31%, three women and one man).

^h^RD: renal disease, (23% of SLE patients).

^i^CRP: C-reactive protein (mg/dL).

^j^N: Neutrophils/microliter.

^k^Dd: Disease duration, at the time of the study, in years (y).

^l^HCQ: hydroxychloroquine; PN: prednisone; MTX: methotrexate; AZA: azathioprine; MMF: mycophenolate mofetil; NT: no treatment.

^m^Median (25th to 75th percentiles).

^n^ *n* = 12.

**Table 2:** Primers for quantitative RT-PCR.

Gene^1^	Accession no.^1^	Description	SABiosciences Cat. no.^2^
IL-1B	NM_000576	Interleukin 1, beta	PPH00171B
IFNG	NM_000619	Interferon, gamma	PPH00380B
IL-12A	NM_000882	Interleukin 12A	PPH00544B
IL-6	NM_000600	Interleukin 6	PPH00560B
TNF-alpha	NM_000594	Tumor necrosis factor (TNF superfamily, member 2)	PPH00341E
IL-10	NM_000572	Interleukin 10	PPH00572B
IL-2	NM_000586	Interleukin 2	PPH00172B
TGFB2	NM_003238	Transforming growth factor, beta 2	PPH00524B
IL-5	NM_000879	Interleukin 5	PPH00692A
TNFSF10	NM_003810	Tumor necrosis factor (ligand) superfamily, member 10	PPH00242E
GAPDH	NM_002046	Glyceraldehyde-3-phosphate dehydrogenase	PPH00150E
ACTB	NM_001101	Actin, beta	PPH00073E
TBX21	NM_013351	T-box-21 (T-bet)	PPH00396A
GATA3	NM_002051	GATA-binding protein 3	PPH02143A
FOXP3	NM_014009	Forkhead box P3	PPH00029B
STAT4	NM_003151	Signal transducer and activator of transcription 4	PPH00777E
RORC	NM_005060	RAR-related orphan receptor C	PPH05877A
MAPK1	NM_002745	Mitogen-activated-protein-kinase 1	PPH00715B
AKT1	NM_005163	v-akt murine-thymoma viral-oncogene-homolog 1	PPH00088A
CD38	NM_001775	CD38 molecule	PPH00856A
ZAP70	NM_001079	Zeta-chain (TCR) associated protein kinase 70 kDa	PPH00256A
CD247	NM_000734	CD247 molecule, CD3-zeta.	PPH01484A
CCR6	NM_004367	Chemokine (C-C motif) receptor 6	PPH00616E

Gene^1,3^	Forward primer (5′–3′)^4^	Reverse Primer (5′–3′)^4^	Ref.

GAPDH (NM_002046)	gaaggtgaaggtcggagtc	gaagatggtgatgggatttc	Primer3 software^3^

^1^ http://www.ncbi.nlm.nih.gov/ .

^2^SABiosciences, QIAGEN company.

^3^ http://frodo.wi.mit.edu/primer3/ .

^4^Primers used for this study based on the literature were computationally checked for sequence specificity. Melt-curve analysis for each primer pair and reaction was also tested to verified specific amplification.

**Table 3:** Cytokines relative mRNA levels in PBMC.

Genes^a^	SLE (*n* = 13)^b^
IL-1B	1.36 (0.75–1.88)
IL-2	1.20 (0.26–6.03)
IL-5	1.41 (0.60–2.70)
IL-6	1.41 (0.58–6.08)
IL-10	1.98^c^ **(1.21–5.37)**
IL-12A	1.67 (0.58–2.81)
IFN-*γ*	1.24 (0.74–2.32)
TNF-*α*	0.20 (0.16–2.20)
TGFB2	1.17 (0.25–2.24)
TNFSF10	1.37 (0.95–1.92)

^a^RNAs extracted from PBMC of SLE patients (*n* = 13) and controls (*n* = 4) were reverse transcribed, and the gene expression levels were determined by qRT-PCR using gene-specific primers for indicated genes to quantify cytokine mRNA expression, by means of a RT^2^ Profiler PCR Array System (Table 2). qRT-PCR methods (Materials and Methods and Supplementary material). Two internal loading gene-specific primers controls were included for standardization between samples (GAPDH and *β*-actin, Table 2). Proportion of transcript present in the samples was calculated using the relative quantification 2^−ΔΔCt^ scheme. Control samples were used as comparative calibrator. Final results represent the relative amount of amplicon in patient's sample (fold change) to the mean level of the transcripts in the control samples.

^b^Values of the relative mRNA levels are presented as Median (25th to 75th percentile).

^c^IL-10 gene expression was increased in SLE patients compared with controls (Wilcoxon Signed Rank test, *P* = 0.0046).

Analysis of the data was done using the GraphPad Prism v.5.01 software.

**Table 4:** Cytokines plasma levels.

Cytokines^a, b^	SLE (*n* = 13)	Controls (*n* = 13)	*P* value^c^
IL-1*β*	0.24 (0.10–0.96)	0.10 (0.10–0.13)	**0.0112**
IL-2	0.61 (0.60–20.24)	0.60 (0.60–2.62)	0.1893
IL-4	0.10 (0.10–0.68)	0.10 (0.10–0.19)	0.6607
IL-5	1.00 (1.00–1.00)	0.90 (0.80–1.00)	**0.0077**
IL-6	1.00 (1.00–22.35)	1.00 (0.85–1.00)	**0.0117**
IL-10	2.82 (0.75–9.64)	0.63 (0.60–3.22)	0.1368
IL-12(p70)	1.02 (0.60–1.68)	0.18 (0.06–0.50)	**0.0009**
IL-13	0.20 (0.20–3.87)	0.20 (0.19–0.21)	0.0685
IFN-*γ*	0.64 (0.20–3.76)	0.20 (0.20–0.33)	**0.0445**
TNF-*α*	0.34 (0.10–2.10)	0.10 (0.10–0.30)	0.1193

^a^A multiplex bead array (Bio-Plex, BioRad) was used to simultaneously measure 10 different cytokines in each plasma sample. Samples from patients and controls were analyzed in parallel. Values are in pg/mL. Limits of detection (pg/mL) for the indicated cytokines were: 0.1, 0.64, 0.17, 0.88, 0.29, 0.17, 0.37, 0.19, 0.35, and 0.2, respectively. Analyses of data were performed using five-parameter logistic curve fitting to standard analyte values. Intra-assay and interassay CV were ≤8% and ≤10%, respectively.

^b^Values are presented as Median (25th to 75th percentiles).

^c^Mann-Whitney *U* test for comparison between SLE and controls.

Significance levels set at *P* values <0.05. The analysis of the data was done using the GraphPad Prism v.5.01 software.

**Table 5:** Spearman's rank correlations obtained between cytokines gene expressions in PBMCs and cytokines plasma levels in SLE patients.

Gene	SLE (*n* = 13)						
	Plasma cytokines						
	IL-1*β*	IL-2	IL-6	IL-10	IL-12(p70)	IFN-*γ*	TNF-*α*
IL-1B							
** ** *r *	0.6082						
** ** *P *	0.0274						
IL-6							
** ** *r *	0.6110	0.7427	0.7204	0.8253	0.6877	0.7856	0.8970
** ** *P *	0.0265	0.0036	0.0055	0.0005	0.0094	0.0015	<0.0001
IL-10							
** ** *r *							0.6101
** ** *P *							0.0268

Gene	Gene						
	IL-2	IL-6		IL-10	IL-12A	IFN-*γ*	TGFB2

IL-2							
*r *	1	0.6538		0.6319	0.9106		0.7868
*P *		0.0153		0.0205	<0.0001		0.0014
IL-6							
*r *		1		0.7582	0.7180		
*P *				0.0027	0.0057		
IL-10							
*r *				1	0.7813		0.6190
*P *					0.0016		0.0241
IL-12A							
*r *					1	0.6905	0.7796
*P *						0.0090	0.0017

Significant Spearman's rank correlations coefficients are indicated.

Significance levels set at *P* values <0.05.

**Table 6:** Spearman's rank correlations found between MAPK1, AKT1, and Th-transcription factors gene expressions in SLE patients.

SLE (*n* = 13)						
Gene	AKT1	T-bet	STAT4	GATA3	RORC	FOXP3
MAPK1						
*r *	0.9188	0.5659		0.5609	0.6154	
*P *	<0.0001	0.0438		0.0463	0.0252	
AKT1						
*r *	1				0.6300	
*P *					0.0210	
T-bet						
*r *		1	0.7912	0.9231	0.8958	0.9066
*P *			0.0013	<0.0001	<0.0001	0.0004
STAT4						
*r *			1	0.7308	0.7253	0.5659
*P *				0.0045	0.005	0.0438
GATA3						
*r *				1	0.9066	0.9066
*P *					<0.0001	<0.0001
RORC						
*r *					1	0.7802
*P *						0.0017

Significant Spearman's rank correlations coefficients are indicated.

Significance levels set at *P* values <0.05.

**Table 7:** Spearman's rank correlations found between gene expressions of AKT1, MAPK1, FOXP3, cytokines, IL-10, and Th-transcription factors.

SLE (*n=*13)						
Gene	AKT1	MAPK1	FOXP3	RORC	GATA3	T-bet
IL-2						
*r *			0.7692			
*P *			0.0021			
IL-5						
*r *	0.6970	0.7895	0.6575			
*P *	0.0081	0.0013	0.0106			
IL-6						
*r *			0.6484			
*P *			0.0165			
IL-10						
*r *		0.5549	0.8022	0.6923	0.7143	0.5604
*P *		0.0490	0.0010	0.0087	0.0061	0.0463
IL-12A						
*r *		0.5942	0.9051			
*P *		0.0322	<0.0001			
IFN-*γ*						
*r *			0.6703			
*P *			0.0122			
TNFSF10						
* r *	0.6556					
*P *	0.0150					
TGFB2						
*r *	0.6584	0.6960	0.6990			
*P *	0.0144	0.0082	0.0082			

Significant Spearman's rank correlations coefficients are indicated.

Significance levels set at *P* values <0.05.

**Table 8:** Spearman's rank correlation test.

SLE (*n* = 13)									
	CRP^c^	N^d^		Age	C4^c^	L^f^	CD38^e^	ZAP70^e^	CCR6^e^
Cytokines^a^			Gene^e^						
IL-1*β*			IL-2						
*r *	0.5944		*r *	0.6658					
*P *	0.0415		*P *	0.0130					
IL-2			IL-10						
*r *	0.6671		*r *	0.6107			0.5915		
*P *	0.0178		*P *	0.0266			0.0332		
IL-6			IL-12A						
*r *	0.6160		*r *	0.6419					
*P *	0.0329		*P *	0.0180					
IL-10			TGFB2						
*r *	0.6690		*r *	0.5909					
*P *	0.0174		*P *	0.0335					
IL-13			TNFSF10						
*r *	0.8629		*r *			−0.5750			
*P *	0.0003		*P *			0.0398			
IFN-*γ*			TNF-*α*						
*r *	0.7261		*r *					0.6740	
*P *	0.0075		*P *					0.0115	
TNF-*α*			AKT1						
*r *	0.6834		*r *				0.6061		
*P *	0.0143		*P *				0.0281		
			GATA-3						
			*r *	0.6190					
			*P *	0.0241					
			STAT4						
			*r *		0.7836				0.5659
			*P *		0.0026				0.0438
Calprotectin^b^			CCR6						
*r *		0.6593	*r *		0.7343				
*P *		0.0142	*P *		0.0065				

^a^Plasma Cytokines (pg/mL).

^b^Calprotectin plasma levels (ng/mL).

^c^ *n* = 12.

^d^N: Neutrophils.

^e^Gene: Gene expression, as indicated in legend of Figure 1.

^f^L: Lymphocytes.

Significant Spearman's rank correlations coefficients are indicated.

Significance levels set at *P* values <0.05.
