# Supplementary material for: Altered AKT1 and MAPK1 Gene Expression on Peripheral Blood Mononuclear Cells and Correlation with T-Helper-Transcription Factors in Systemic Lupus Erythematosus Patients
Source: Mediators Inflamm. 2012 Oct 18;2012:495934. doi: 10.1155/2012/495934 (PMC3483815; doi:10.1155/2012/495934)
Supplement: Supplementary file 1 — qRT-PCR procedures: Reverse transcription (RT) reaction was performed on 1 µg total RNA, 20-µl reaction, containing 200 U SuperScript III reverse transcriptase (Invitrogen), 5 mM random hexamer primers, 0.5mM each deoxyribonucleotide triphosphate (dNTP), 20 U RNase inhibitor, and 1X-RT buffer. Primer sequences and gene-specific primers used are available in Table 2. Gene expressions assays were performed on iQ-Cycler (Bio-Rad), with 3 replicates with iQ-SYBR Green Supermix, 21-microliter reaction (200nM each primer and 10 ng of cDNA). PCR program: 10 min incubation at 95°C, 40 cycles of 15 s at 95°C, and 1 min at 60°C. Specificity of PCR amplification procedure was checked with a heat dissociation protocol (from 65°C to 100°C), after the final cycle of the PCR. [file 495934.f1.pdf]

Supplementary Table 1 Test to analyze the Gaussian distribution for cytokines plasma levels in SLE patients. [1][Kolmogorov-Smirnov (KS) test (with Dallal-Wilkinson-Lilliefor P value]

	IL-1β	IL-2	IL-4	IL-5	IL-6	IL-10	IL-12	IL-13	IFN-γ	TNF-α
KS normality test										
KS distance	0.3467	0.2942	0.3782	0.4866	0.3726	0.2319	0.1984	0.3056	0.2623	0.3159
P value	0.0001	0.0030	P<0.0001	P<0.0001	P<0.0001	0.0542	P > 0.10	0.0016	0.0148	0.0009
Passed normality test (alpha=0.05)?	No	No	No	No	No	Yes	Yes	No	No	No
P value summary	***	**	***	***	***	ns	ns	**	*	***
D'Agostino & Pearson omnibus normality test										
K2	4.246	5.512	20.04	29.18	16.75	8.592	14.61	4.113	3.481	3.291
P value	0.1197	0.0635	P<0.0001	P<0.0001	0.0002	0.0136	0.0007	0.1279	0.1755	0.1929
Passed normality test (alpha=0.05)?	Yes	Yes	No	No	No	No	No	Yes	Yes	Yes
P value summary	ns	ns	***	***	***	*	***	ns	ns	ns
Shapiro-Wilk normality test										
W	0.6847	0.7518	0.5787	0.4290	0.6059	0.7518	0.8044	0.7600	0.8080	0.7276
P value	0.0004	0.0019	P<0.0001	P<0.0001	P<0.0001	0.0019	0.0077	0.0024	0.0085	0.0011
Passed normality test (alpha=0.05)?	No	No	No	No	No	No	No	No	No	No
P value summary	***	**	***	***	***	**	**	**	**	**

Supplementary Table 2 Test to analyze the Gaussian distribution for cytokines plasma levels in Controls. [1][Kolmogorov-Smirnov (KS) test (with Dallal-Wilkinson-Lilliefor P value]

	IL-1β	IL-2	IL-4	IL-5	IL-6	IL-10	IL-12	IL-13	IFN-γ	TNF-α
KS normality test										
KS distance	0.4100	0.4136	0.3568	0.5060	0.4778	0.2882	0.2401	0.5204	0.3797	0.2666
P value	P<0.0001	P<0.0001	P<0.0001	P<0.0001	P<0.0001	0.0042	0.0391	P<0.0001	P<0.0001	0.0121
Passed normality test (alpha=0.05)?	No	No	No	No	No	No	No	No	No	No
P value summary	***	***	***	***	***	**	*	***	***	*
D'Agostino & Pearson omnibus normality test										
K2	26.26	9.722	7.196	35.13	25.63	7.241	14.46	35.87	15.92	9.546
P value	P<0.0001	0.0077	0.0274	P<0.0001	P<0.0001	0.0268	0.0007	P<0.0001	0.0003	0.0085
Passed normality test (alpha=0.05)?	No	No	No	No	No	No	No	No	No	No
P value summary	***	**	*	***	***	*	***	***	***	**
Shapiro-Wilk normality test										
W	0.5786	0.5927	0.7116	0.3951	0.4820	0.7643	0.7490	0.3487	0.5575	0.8162
P value	P<0.0001	P<0.0001	0.0007	P<0.0001	P<0.0001	0.0026	0.0018	P<0.0001	P<0.0001	0.0107
Passed normality test (alpha=0.05)?	No	No	No	No	No	No	No	No	No	No
P value summary	***	***	***	***	***	**	**	***	***	*

Supplementary Table 3 Test to analyze the Gaussian distribution for cytokines relative mRNA levels in PBMC. [1][Kolmogorov-Smirnov (KS) test (with Dallal-Wilkinson-Lilliefor P value]

	IL1B	IFNG	IL12A	IL6	TNF	IL10	IL2	TGFB2	IL5	TNFSF10
[1]KS normality test										
KS distance	0.2147	0.2170	0.2549	0.2407	0.2667	0.2540	0.2923	0.2339	0.3456	0.1720
P value	P > 0.10	0.0954	0.0207	0.0381	0.0121	0.0216	0.0034	0.0500	0.0006	P > 0.10
Passed normality test (alpha=0.05)?	Yes	Yes	No	No	No	No	No	Yes	No	Yes
P value summary	ns	ns	*	*	*	*	**	ns	***	ns
D'Agostino & Pearson omnibus normality test										
K2	5.590	17.04	1.675	8.021	17.68	2.954	23.63	2.914	23.02	1.911
P value	0.0611	0.0002	0.4328	0.0181	0.0001	0.2283	P<0.0001	0.2329	P<0.0001	0.3847
Passed normality test (alpha=0.05)?	Yes	No	Yes	No	No	Yes	No	Yes	No	Yes
P value summary	ns	***	ns	*	***	ns	***	ns	***	ns
Shapiro-Wilk normality test										
W	0.8835	0.7856	0.8693	0.8169	0.7106	0.8486	0.6353	0.8630	0.6046	0.9266
P value	0.0794	0.0046	0.0512	0.0109	0.0007	0.0274	0.0001	0.0422	P<0.0001	0.3072
Passed normality test (alpha=0.05)?	Yes	No	Yes	No	No	No	No	No	No	Yes
P value summary	ns	**	ns	*	***	*	***	*	***	ns
